# Health‐Related Quality of Life and Social Reintegration Indicators Following Reconstructive Surgery: A Prospective Observational Study

**DOI:** 10.1002/wjs.12696

**Published:** 2025-07-09

**Authors:** Shireen Dumont, Silas Msangi, Simon Ponthus, Leslie Elahi, Roba Khundkar, Edward Wayi, Lionel Dumont

**Affiliations:** ^1^ Division of Dermatology and Venereology Geneva University Hospitals Geneva Switzerland; ^2^ 2^ND^ Chance Association Geneva Switzerland; ^3^ Department of Surgery Tumbi Regional Referral Hospital Kibaha Tanzania; ^4^ Division of Anaesthesiology Department of Acute Care Medicine Geneva University Hospitals Geneva Switzerland; ^5^ Department of Plastic and Reconstructive Surgery Institut Curie Paris France; ^6^ Department of Plastic Surgery Sheffield Teaching Hospitals NHS Foundation Trust Sheffield UK; ^7^ Department of Anaesthesiology Pharmacology Intensive Care and Emergency Medicine Faculty of Medicine University of Geneva Geneva Switzerland

**Keywords:** health‐related quality of life, low‐ and middle‐income countries, patient‐reported outcome measures, reconstructive surgery, short‐term surgical mission

## Abstract

**Background:**

Studies focusing on disease severity and reconstructive surgical treatment's impact on health‐related quality of life (HRQOL) are lacking, particularly in low‐ and middle‐income countries (LMICs). This study aims to assess the impact of reconstructive surgery‐related conditions on basic indicators of quality of life and social integration within the context of limited resources and short‐term surgical missions.

**Methods:**

We conducted a pre‐post cohort study at Tumbi Regional Referral Hospital in Tanzania from July 2023 to July 2024. Patients undergoing reconstructive surgery for postburn contractures (PBC), congenital malformations, and trauma‐related conditions were included. Surgical outcomes and HRQOL were assessed using patient‐reported outcome measures (PROMs) preoperatively and at 12 months postoperatively.

**Results:**

Of 120 scheduled patients, 82 were included, with a 12‐month follow‐up rate of 77.7% and a median age of 3.5 years old. PBC accounted for 73.2% of cases. The patients' primary expectation after surgery was functional recovery (64%). Patient‐reported disabilities improved significantly, decreasing from 72% to 9% postoperatively (*p* < 0.001). The impact on family life improved from 58.8% to 6% (*p* < 0.001), and reductions in social exclusion and discrimination were observed. Notably, perceptions of witchcraft association declined from 23% to 7.8% (*p* < 0.014).

**Conclusions:**

Assessing the impact of disabilities and surgical outcomes on HRQOL using PROMs is feasible and seems essential during short‐term surgical missions. The findings suggest that reconstructive surgery restores functionality and improves quality of life 1 year after the procedure, highlighting its positive impact on patients' social lives and overall well‐being.

## Graphical abstract

Studies focusing on disease severity and reconstructive surgical treatment's impact on health‐related quality of life (HRQOL) are lacking, particularly in low‐ and middle‐income countries (LMICs). This study aims to assess the impact of reconstructive surgery‐related conditions on basic indicators of quality of life and social integration within the context of limited resources and short‐term surgical missions. We conducted a pre‐post cohort study at Tumbi Regional Referral Hospital in Tanzania from July 2023 to July 2024.

## 1 Introduction

Surgical needs are substantial in low‐ and middle‐income countries (LMICs), and common indications for reconstructive surgery include burn sequelae, traumatic injuries, and congenital malformations (e.g., syndactyly, amniotic band syndrome, clefts) [1, 2].

Surveys from several countries in sub‐Saharan Africa report a lack of surgical capacities and especially subspecialized physicians, including plastic surgeons [3].

Reconstructive surgery aims to restore a person's integrity by repairing or reshaping a mutilated or deformed part of the body with both functional and esthetic goals [2, 4, 5, 6]. Left untreated, these pathologies may result in severe functional and esthetic disabilities, significantly impacting quality of life through social exclusion, feelings of shame for families, and local beliefs such as accusations of witchcraft [7, 8, 9]. Studies focusing on disease severity and reconstructive surgical treatment's impacts on health‐related quality of life (HRQOL) in LMICs are scarce for many reasons [10, 11, 12]. First, pathologies differ significantly between high‐income countries (HICs) and LMICs. A notable example is postburn contractures (PBC). Indeed, LMICs are disproportionately affected by burns, with very few resources to prevent or treat burn scar contractures, especially in low‐income countries. This leads to delays in care or no care at all, resulting in disfiguring outcomes and limited range of joint motion, which severely impacts quality of life [9, 12]. Secondly, surgically validated standardized measures of HRQOL are missing or designed for HICs, lacking consideration for local social determinants of health, difficulties accessing care, and cultural stigma in LMICs. Most of the research in this field has been done for cleft lip and palate surgery [10, 11, 13, 14], always emphasizing the need to identify and create an accurate patient‐reported outcome measure (PROMs) for this population.

This study aims to assess the impact of reconstructive surgery‐related conditions on basic indicators of quality of life and social integration within the context of limited resources and regular short‐term surgical missions. The primary objective of this study is to compare simple indicators of quality of life and social integration before surgery and 1 year afterward. The secondary objective is to assess whether surgical outcomes, pathologies, and expectations influence quality of life indicators. The third objective is to demonstrate that regular short‐term reconstructive surgery missions can meet standard criteria for follow‐up quality.

## 2 Methods

### 2.1 Study Design

This was a prospective, longitudinal, monocentric cohort study conducted at Tumbi Regional Referral Hospital in Tanzania. It was carried out by the 2nd Chance Association, a nonprofit organization operating primarily in sub‐Saharan African countries, during a short‐term reconstructive surgery mission [15]. Patients were enrolled between July 2023 and July 2024.

All patients scheduled for reconstructive surgery (sequelae of burns, congenital malformation, and accidents with physical impact) were included in this study. Patients unable to give consent or whose parents did not give their consent were excluded.

All patients signed a consent form. Ethical committee approval was obtained from the College of Surgeons of East, Central and Southern Africa (COSECSA) board. All participants were provided with a consent form, along with oral and written information in both English and Kiswahili, outlining this study and offering adequate details to enable them to make an informed decision about their participation.

### 2.2 Setting Up the Questionnaire and the Follow‐Up

All patients presenting on the screening day were evaluated. Surgical selection was based on compatibility with mission resources: relevant indication, no need for unavailable diagnostic tests (e.g., MRI), absence of high‐risk comorbidities, and no requirement for advanced anesthesia or high hemorrhagic‐risk procedures.

At the end of the mission, all patients were reassessed and discharged with postoperative care instructions. Follow‐up evaluations were conducted by the local team at 1 week, 4 weeks, and 12 weeks to manage potential complications or prescribe physiotherapy if needed. A follow‐up visit was organized at 1 year to assess the final surgical outcomes.

To maximize patient participation in the follow‐up, an incentive system was implemented. This system compensated the local surgical team for their additional workload and encouraged patients to return for postoperative visits.

Data collection was conducted preoperatively on the day before surgery and again 1 year later. Preoperative and postoperative photographs and pathology descriptions were collected for each patient. The social follow‐up included a two‐part survey assessing quality of life and expectations: the first part was completed during preoperative screening, and the second one, 1 year after surgery. The 1‐year interval was chosen as it aligned with the timing between two missions and represented the period when the most significant improvements in quality of life are typically observed [9].

The surgical outcomes and impact on HRQOL were assessed using a questionnaire of five questions, drawing from multiple PROMs [16], such as the WHO Disability Assessment Schedule (WHODAS 2.0) [17], Participation Scale (P‐scale) [18], and Burn Specific Health Scale‐Brief (BSHS‐B) [19]. These questions were chosen and tested during the pilot phase of this study to evaluate their relevance, clarity, and cultural appropriateness. This initial testing allowed us to refine the questions, combining standardized instruments, ensuring they effectively capture the social and functional impact of the condition while remaining understandable and applicable to the target population. Questions were translated with the assistance of an English‐Kiswahili translator. As most patients were children under the age of 12, their parents acted as proxies, completing the questionnaires on their behalf.

These questions were asked identically both before and 1 year after the operation.

1. Disability: Do you have any difficulties in daily life because of the disability related to surgery? (yes or no)
2. Family: Does the pathology have any impact on family life? (yes or no)
3. Exclusion: Do you experience any kind of exclusion from your community? (yes or no)
4. Discrimination: Do you feel any kind of discrimination related to your disability? (yes or no)
5. Witchcraft: Is your disability considered by some to be a form of witchcraft? (yes or no)

Patients were asked preoperatively about their expectations (aesthetic, functional, or social—e.g., return to work or school). Postoperatively, they indicated whether these expectations were met. Surgical outcomes were independently assessed by two expert plastic surgeons (not necessarily the operators) and classified as good, partial, or failed.

### 2.3 Stratification by Pathologies Related to Reconstructive Surgery

Based on experiences from previous missions, reconstructive surgery‐related pathologies were categorized into six groups: PBC, congenital hand and foot deformities (e.g., syndactyly), keloid scars, sequelae of accidents (e.g., road accidents, animal bites), clefts, and cancers.

### 2.4 Statistical Analysis Plan

This study included all patients who participated at baseline (preoperative assessment), with missing data for 20% of the participants (*n* = 18) at the 1‐year postoperative follow‐up. We compared outcomes between baseline and 1‐year follow‐up for the overall sample and conducted stratified analyses by reconstructive surgery pathologies and surgical outcomes. Pearson's chi‐squared test and Fisher's exact test were used for comparisons of categorical variables, when appropriate. Statistical significance was set at *p* < 0.05 (two‐sided), and all analyses were performed using R (version 4.4.0). Missing data (MD) are indicated in the tables and most often reflect questions that patients either declined to answer or did not fully understand. Longitudinal analyses were performed on complete cases only; participants with missing data were excluded.

## 3 Results

### 3.1 Demographic Data

**FIGURE 1:**
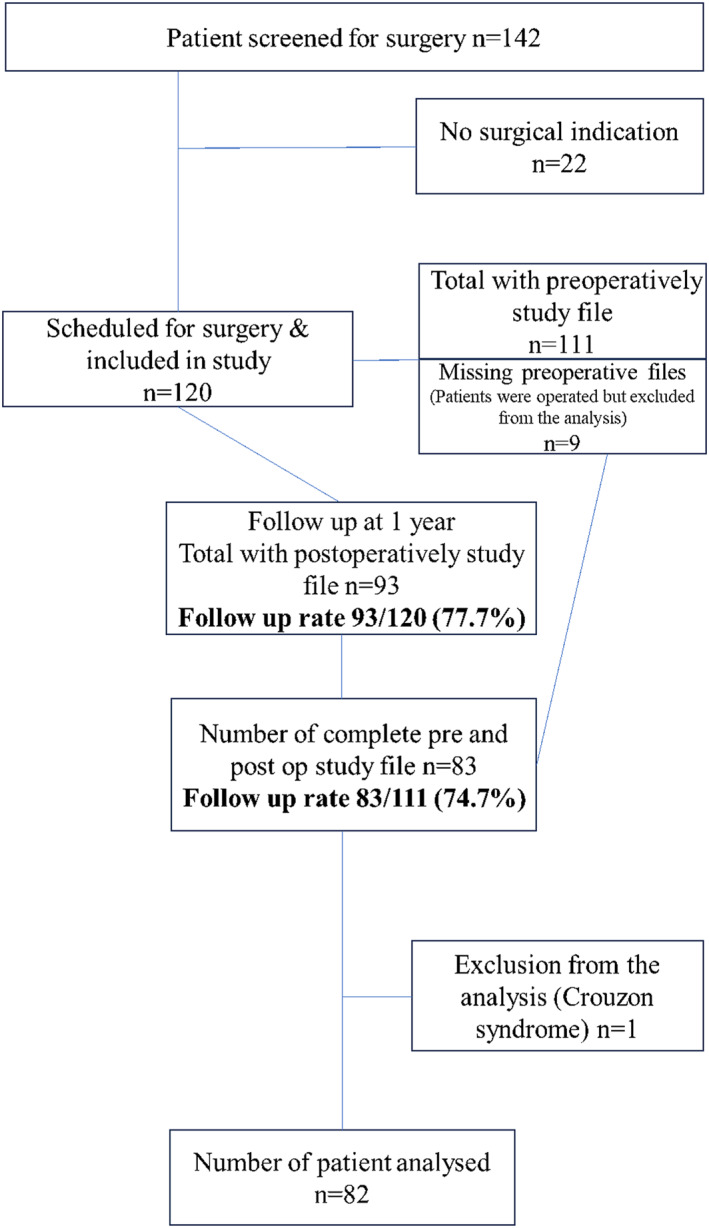
Flow chart of patients included and analyzed in this study.

Out of 142 patients, 120 were scheduled for surgery and enrolled in this study. Among them, 82 were fully analyzed (Figure 1). A patient with Crouzon syndrome was excluded from this study, considering that the consequences of this condition could not be corrected solely with reconstructive surgery. The follow‐up rate was 77.7% at 1 year, and the results were ultimately analyzed on 74.7% of the patients (*n* = 82) with complete records and questionnaires.

Patients' characteristics are presented in Table 1. The median age of the patients was 3.5 years, ranging from 1 to 44 years. The sex ratio was equal.

**TABLE 1:** Demographic data of number of patients analyzed and number of patients able to answer all questions.

Characteristics	Number of patients analyzed
Demographic data	*n* = 82
Age (median) [IQR]	3.5 [4.8]
M/F	41/41
> 16 years	*n* = 12 (mean 26 +/−7)//25
M/F > 16	6F/6M
< 16 years	*n* = 70 (mean 4+/−3)//3

Abbreviation: IQR, interquartile range.

The surgery groups related to reconstructive surgery were separated in three groups (Table 2): the PBC group being the more prevalent with 73.2% of participants (*n* = 60), malformations such as syndactyly of the upper and lower limbs (*n* = 15), and other pathologies (*n* = 7) among them: clefts (*n* = 1), keloid scars (*n* = 2), post‐trauma scars (*n* = 3), and a sequela of compartment syndrome (*n* = 1). Among the highest expectations after surgery, recovering functionality was the most reported (64%). Surgical outcome was considered successful in 73% of cases, and 95% of the participants were satisfied by surgery.

**TABLE 2:** Surgery: Characteristics, expectations, and outcomes.

Surgery: Characteristics, expectations, and outcomes	Number of patients analyzed
Pathologies	
Postburn contracture	*n* = 60 (73.2%)
Congenital hand and foot deformities	*n* = 15 (18.3%)
Other	*n* = 7 (8.5%)
Expectation from surgery	
Recovery of functionality	53/82 (64%)
Improved aesthetics	32/82 (39%)
Back to work/back to school	11/82 (13%)
Back to normal life	23/82 (28%)
Surgical outcome (evaluated by surgeon)	
Good (meets surgical expectation)	60 (73%)
Partially (meets surgical expectation partially)	18 (22%)
Failure	4 (5%)
Surgical outcome (evaluated by patient) with the question: Are you satisfied by the surgery?	
Yes	78 (95%)
No	4 (5%)^a^

^a^ Among the patients where surgery failed, 4/4 (100%) were unsatisfied.

### 3.2 Patient‐Reported Outcomes on Disability, Impact on Family Life, Exclusion, Discrimination, and Witchcraft Consideration Before and After Surgery

Among patient‐reported outcomes (Table 3), 72% of patients reported difficulties in daily life related to disability before surgery versus 9% (*p* value < 0.001) after surgery; 58.8% reported an impact on family life before surgery versus 6% (*p* value < 0.001) after surgery; 51.2% reported any kind of exclusion before surgery versus 7% (*p* value < 0.001) after surgery; 62% reported any kind of discrimination before surgery versus 18% (*p* value < 0.001) after surgery; and 23% were considered to have witchcraft before surgery versus 7.8% (*p* value < 0.014) after surgery.

**TABLE 3:** Longitudinal analysis: Comparison of indicators between before surgery and 1 year after surgery on the overall sample.

*n* = 82 patients	Preop *n* %	MD	Post op *n* %	MD	*p* value^a^
Disability	59	0	7	0	< 0.001
	72.0%		9%		
Family	48	0	5	2	< 0.001
	58.8%		6%		
Exclusion	42	0	6	3	< 0.001
	51.2%		7%		
Discrimination	51	0	15	3	< 0.001
	62%		18%		
Witchcraft	17	9	5	18	0.014
	23%		7.8%		

*Note: n* (%); Disability: *n* patients with any difficulties in daily life because of the disability related to surgery. Family: *n* patients having any impact on family life?. Exclusion: *n* patients with experience of any kind of exclusion from your community?. Discrimination: *n* patients with experience of any kind of discrimination related to disability. Witchcraft: *n* patients considered to have witchcraft related to disability.

Abbreviation: MD = missing data.

^a^ Pearson's chi‐squared test on complete cases.

### 3.3 Subgroup Analysis

Within the subgroup analysis (Supporting Information S1 and Table 4), similar trends were observed in the PBC group (Supporting Information S1): 74% of patients reported difficulties in daily life due to disability before surgery compared to 10% after surgery (*p* < 0.001); 59% reported an impact on family life before surgery versus 6.9% after surgery (*p* < 0.001); 57% experienced some form of exclusion before surgery versus 8.6% after surgery (*p* < 0.001); 64% reported experiencing discrimination before surgery versus 13% after surgery (*p* < 0.001); and 22% were considered to have witchcraft before surgery compared to 4.3% after surgery (*p* < 0.01). For congenital hand and foot deformities and other conditions, statistical power was limited due to the relatively small sample size.

**TABLE 4:** Comparison of indicators between before surgery and 1 year after surgery—stratified by outcome of surgery.

*n* = 82	Good (*n* = 60)					Partial (*n* = 18)					Failure (*n* = 4)				
	Preop *n* %	MD	Post op *n* %	MD	*p* value^a^	Preop *n* %	MD	Post op *n* %	MD	*p* value^b^	Preop *n*%	MD	Post op *n* %	MD	*p* value^c^
Disability	43	0	4	1	< 0.001	13	0	0	0	< 0.001	3	0	3	0	> 0.9
	72%		6.8%			72%		0%			75%		75%		
Family	37	0	3	3	< 0.001	9	0	1	0	0.003	2	0	1	0	> 0.9
	62%		5.3%			50%		5.6%			50%		25%		
Exclusion	36	0	4	5	< 0.001	6	0	2	0	0.2	0	0	0	0	n/a
	60%		7%			33%		11%			0%		0%		
Discrimination	41	0	9	4	< 0.001	8	0	5	0	0.3	2	0	1	0	> 0.9
	68%		16%			44%		28%			50%		25%		
Witchcraft	14	6	5	12	0.045	2	3	0	5	0.5	1	0	0	1	> 0.9
	26%		10%			13%		0%			25%		0%		

*Note:* Disability: *n* patients with any difficulties in daily life because of the disability related to surgery. Family: *n* patients having any impact on family life?. Exclusion: *n* patients with experience of any kind of exclusion from your community?. Discrimination: *n* patients with experience of any kind of discrimination related to disability. Witchcraft: *n* patients considered to have witchcraft related to disability.

Abbreviations: MD = missing data; *n* = number of patients; % = percentage of patients.

^a^ Pearson's chi‐squared test.

^b^ Pearson's chi‐squared test; Fisher's exact test.

^c^ Fisher's exact test on complete cases.

In the subgroup analysis stratified by surgical outcomes (Table 4), the trends indicating improvements across our five items remain similar when the surgical result is considered good or partial. However, when surgery fails, no changes have been observed.

The demographic data and surgical characteristics are summarized in Table 1.

## 4 Discussion

We prospectively followed 82 patients in this study, the majority of whom were suffering from PBC and congenital malformations (such as syndactyly). The findings suggest that reconstructive surgery helps restore functionality and leads to improvements in quality‐of‐life indicators 1 year after the procedure. This underscores the significant impact of certain disfiguring pathologies on patients' social lives and highlights the potential of reconstructive surgery to enhance their overall quality of life.

The first noteworthy observation is the extent of social suffering these conditions cause prior to surgery. Over 70% of patients experienced issues in their family lives, more than half experienced social exclusion and discrimination, and more than 20% endured stigma associated with beliefs such as witchcraft due to their conditions. Our findings seem in line with this recent study that aimed to assess the development of burn scar contractures and their impact on joint function, disability, and quality of life at 1‐year follow‐up, concluding that burn scar contractures were highly associated with disability and low HRQOL [20].

The second key observation is that, in this study, surgery significantly improves these circumstances after 1 year, particularly when the surgical outcomes are positive (e.g., recovery of function, esthetic results). However, suffering indicators remain with an incidence between 6% and 18% at the 12‐month follow‐up point.

Improvements in quality‐of‐life scores have been demonstrated previously [9, 21]. Jenkinson et al. describe a significant reduction in social isolation after facial surgery, albeit to a lesser extent among older patients, highlighting the greater difficulty in reintegrating into the community with advancing age. Similarly, in our experience, we found a comparable trend among adolescents and adults compared to children.

Hendriks et al. conducted a recent longitudinal study focusing on functional impact on PBC release surgery in LMICs and reported favorable outcomes in terms of range of motion, disability, and HRQOL at 1‐year follow‐up [9]. One major limitation reported in these studies mentioned that questionnaires used were only partially relevant in the setting of sub‐Saharan African countries and for a pediatric population [9, 20].

The strengths of this study lie in its evaluation of surgery within the context of an LMIC and its high follow‐up rate. With a follow‐up rate of 77.7%, this study aligns with the highest 1‐year rates reported in reconstructive surgery missions for cleft repair and PBC [12]. However, it is concerning that 22% of the patients were lost to follow‐up after 1 year. Considering the generally poor surgical outcomes reported for LMICs [22], it is crucial to investigate whether these patients are still alive, whether the intervention was successful, or the reasons for their absence from the 1‐year follow‐up, regardless of the outcome. Potential barriers include the distance between patients' homes and the hospital, their occupations, and their social circumstances (whether related to or independent of their surgical condition) [12].

Our study has certain limitations. There is no consensus on the most suitable PROMs for patients undergoing reconstructive surgery (e.g., burn survivors as an example), particularly in the context of LMICs [23]. As a result, and due to constraints of the mission setting, including limited time, a high volume of patients to assess, and the limited availability of translators, we had to select questions based on their feasibility, focusing on the impact of pathologies on quality of life and social life indicators, as well as surgical outcomes. Questions were selected based on consensus after discussions with local surgeons and healthcare workers, as well as with the involvement of patients themselves. The question regarding witchcraft was specifically included after conversations with patients, as witchcraft was frequently mentioned as a major factor contributing to their stigmatization.

Although the questions were inspired by validated tools, we were unable to compare our simplified five‐item questionnaire with a more comprehensive and validated instrument. During the pilot phase, it soon became evident that using standardized QoL scales with formal scoring systems was not feasible in our setting due to significant language barriers and limited time available for each interview. Consequently, one of the unique aspects of this study is the dichotomous approach to measuring patients' perception of suffering and quality of life using closed‐ended questions. It may have reinforced disparities in how patient experiences are documented across different global settings or introduced a bias by overestimating the positive effect of surgery on interventions whose outcomes were only partially successful. However, although this method does not capture the subtle nuances of feelings, it effectively highlights the prevalence of poor quality of life and its improvement after surgery.

As pointed out in other studies [9, 20], questions were answered by proxies on behalf of the children, necessitating careful interpretation of the results. Despite the small sample size, the subgroup analyses consistently demonstrate that surgery improves indicators in nearly all cases, regardless of the population, pathologies, expectations, or outcomes.

Immediate complications were not reported in this study. This does not imply their absence but rather indicates that, if any occurred, they were managed by the local teams. Consequently, their incidence, progression, or impact on the final surgical outcomes were not reported.

Finally, by offering payment for participants to return and answer questions, we may have introduced bias, either in terms of participation or a tendency to provide more positive responses.

This study also underscores the feasibility of conducting rigorous outcome assessments for regular short‐term reconstructive surgery missions [24]. Achieving this requires infrastructure, financial investment, collaboration with local stakeholders [25], and returning to the same location annually [12]. These missions also serve as training workshops for young African surgeons, making the accurate assessment of long‐term surgical outcomes a valuable educational component that informs their future practice.

Our study highlights the impact of reconstructive surgical missions not only on surgical outcomes and the restoration of functional motion, but also on improving patients' quality of life. Further studies with larger samples are needed to more thoroughly analyze the social impact of surgery performed during short‐term surgical missions in low‐resource settings.

## Author Contributions

**Shireen Dumont:** conceptualization (lead), writing – original draft (lead). **Silas Msangi:** project administration, resources (lead), writing – review and editing (equal). **Simon Ponthus:** writing – review and editing (equal). **Leslie Elahi:** writing – review and editing (equal). **Roba Khundkar:** writing – review and editing (equal). **Edward Wayi:** project administration, resources (equal). **Lionel Dumont:** conceptualization (lead), methodology (lead), formal analysis (lead), writing – review and editing (equal).

## Disclosure

The authors have no financial interest to declare in relation to the content of this article.

## Consent

Informed consent was obtained from all individual participants included in this study.

## Conflicts of Interest

The authors declare no conflicts of interest.

## Supporting information

Supporting Information S1

## Data Availability

The data that support the findings of this study are available from the corresponding author upon reasonable request.
