# Supplementary material for: Health‐Related Quality of Life and Social Reintegration Indicators Following Reconstructive Surgery: A Prospective Observational Study
Source: World J Surg. 2025 Jul 9;49(10):2794–800. doi: 10.1002/wjs.12696 (PMC12515029; doi:10.1002/wjs.12696)
Supplement: Supplementary file 1 — Supporting Information S1 [file WJS-49-2794-s001.docx]

Appendix 1: Comparison of indicators between before surgery and one year after surgery-stratified by pathologies related to reconstructive surgery

| n=82 | **Post-Burn contracture (n=60)** | | | | | **Congenital hand and foot deformities (n=15)** | | | | | **Other (n=7)** | | | | |
| --- | --- | --- | --- | --- | --- | --- | --- | --- | --- | --- | --- | --- | --- | --- | --- |
|  | **Preop**  **n**  **%** | **MD** | **Post op**  **n**  **%** | **MD** | **P value*^1^*** | **Preop**  **n**  **%** | **MD** | **Post op**  **n**  **%** | **MD** | **P value*^2^*** | **Preop**  **n**  **%** | **MD** | **Post op**  **n**  **%** | **MD** | **P value*^3^*** |
| **Disability** | 45  74% | 0 | 6  10% | 1 | <0.001 | 8  57% | 0 | 1  7% | 0 | 0.013 | 6  86% | 0 | 0  0% | 0 | 0.05 |
| **Family** | 36  59% | 0 | 4  6.9% | 3 | <0.001 | 7  50% | 0 | 0  0% | 0 | 0.006 | 5  71% | 0 | 1  14% | 0 | 0.10 |
| **Exclusion** | 35  57% | 0 | 5  8.6% | 3 | <0.001 | 4  29% | 0 | 0  0% | 1 | 0.10 | 3  43% | 0 | 1  17% | 1 | 0.6 |
| **Discrimination** | 39  64% | 0 | 8  13% | 3 | <0.001 | 7  50% | 0 | 5  36% | 0 | 0.4 | 5  71% | 0 | 2  33% | 1 | 0.3 |
| **Witchcraft** | 22  22% | 6 | 2  4.3% | 14 | 0.010 | 0  0% | 0 | 1  9.1 | 1 | 0.5 | 5  83% | 1 | 2  33% | 1 | 0.2 |

MD= missing data; n=number of patients; %=percentage of patients; *^1^* Pearson’s Chi-squared test; *^2^* Pearson’s Chi-squared test; Fisher’s exact test; *^3^* Fisher’s exact test, on complete cases

Disability: n patients with any difficulties in the daily life because of the disability related to surgery

Family: n patients having any Impact on family life?

Exclusion: n patients with experience of any kind of Exclusion from your community?

Discrimination: n patients with experiencing Any Kind of Discrimination related to disability

Witchcraft: n patients to have witchcraft related to disability
